# Spatial Imbalance of Innate-like T-Cell Niches Underlies Clinical Trajectories in Psoriasis

**DOI:** 10.3390/ijms27020715

**Published:** 2026-01-10

**Authors:** Caio Santos Bonilha

**Affiliations:** Institute of Infection, Immunity and Inflammation, University of Glasgow, Glasgow G12 8TA, UK; caio.bonilha@glasgow.ac.uk

**Keywords:** spatial transcriptomics, iLTC, γδ T cell, MAIT cell, autoimmune disease

## Abstract

Innate-like T cells (iLTCs) are rapid sentinels at epithelial surfaces, yet their spatial organisation and tissue-linked programmes in psoriatic inflammation remain incompletely defined. Spatial transcriptomics from independent cohorts maps γδT and mucosal-associated invariant T cells (MAIT) niches across psoriatic skin and reveals sharply divergent skin-layer arrangements. Psoriatic plaques show expansion of both niches, with γδT transcriptional signatures present in dermis and epidermis and MAIT signatures strongly enriched in the epidermis. Their compartment-specific positioning is mirrored by distinct transcriptional activities that support dermal-sentinel behaviour for γδT-enriched niches and epithelial-retention programmes for MAIT niches. Clinical severity associates with opposite niche dynamics, marked by decreasing dermal γδT frequencies and increasing epidermal MAIT abundance. Functional profiles reinforce this divergence, as dermal γδT niches display rising exhaustion-associated features with greater severity, whereas epidermal MAIT niches show stronger inflammatory and proliferation-related signals. Peripheral CITE-seq profiling identifies parallel systemic patterns, with reduced γδT frequencies and increased MAIT frequencies in blood, along with exhaustion-associated features in γδT cells and MAIT-specific trafficking cues that align with their behaviour in psoriatic tissue. Together the findings define a spatially imbalanced γδT–MAIT axis in psoriatic inflammation that is linked to layer-specific organisation to local inflammatory cues, systemic immune engagement and clinical severity.

## 1. Introduction

Psoriatic inflammation reshapes the skin barrier through sustained cytokine signalling and structural perturbation [1]. Within this environment, innate-like T cells (iLTCs), including γδT cells and mucosal-associated invariant T cells (MAIT) cells, contribute to local immune responses as rapid sentinel populations positioned at the barrier surface [2,3]. Although both subsets have been linked to psoriatic pathology, their organisation across lesional compartments and the programmes associated with their tissue niches remain insufficiently defined.

Spatial transcriptomics situates immune populations within intact tissue architecture allowing microenvironmental influences to be interpreted alongside spatial context [4,5]. This framework allows iLTCs to be viewed in relation to the epithelial and stromal structures that form their immediate surroundings. In psoriatic lesions, resolving iLTC signatures in situ may reveal whether these subsets occupy distinct tissue compartments and how such positioning relates to the molecular activities that define their tissue behaviour.

Through the integration of tissue-resolved transcriptomic maps with peripheral immune profiling across the psoriatic spectrum, this work delineates the organisation of iLTC niches and the programmes that accompany their localisation. A coherent picture emerges in which γδT and MAIT niches display divergent epidermal and dermal organisation linked to clinical severity, with peripheral findings showing related alterations in individuals with greater systemic inflammatory engagement.

## 2. Results

### 2.1. Psoriatic Lesions Exhibit Enriched γδT and MAIT Niches with Signs of Divergent Tissue Programmes

**Figure 1:**
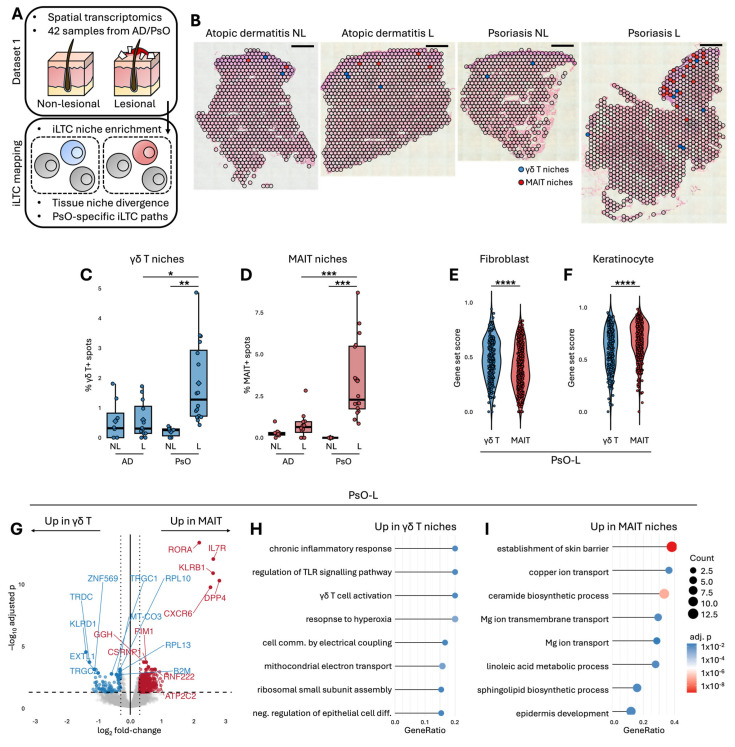
Spatial mapping identifies innate-like T-cell-enriched niches in psoriatic lesions. (**A**) Overview of the spatial transcriptomic analysis workflow. (**B**) Representative histology images overlaid with Visium spot maps. Scale bar = 500 µm. (**C**) Percentages of γδT- or (**D**) MAIT-enriched spots. (**E**) Fibroblast and (**F**) keratinocyte signature scores. (**G**) Differential expression analysis between γδT- and MAIT-enriched PsO-L spots. Vertical dashed lines indicate the fold-change threshold for differential expression, and the horizontal dashed line denotes the significance cutoff corresponding to an adjusted *p* value of 0.05. (**H**) Gene-ontology enrichment of pathways upregulated in γδT- and (**I**) MAIT-enriched niches showing top biological processes by GeneRatio. All spatial transcriptomic analyses shown refer to ILTC-enriched niches defined at the Visium spot level and reflect niche-level transcriptional contexts rather than cell-intrinsic states or composition. Kruskal–Wallis with Wilcoxon rank-sum tests were applied. Student’s *t*-test was used in panels (**E**,**F**). * *p* < 0.05, ** *p* < 0.01, *** *p* < 0.001, **** *p* < 0.0001.

Spatial transcriptomic sections from atopic dermatitis (AD) and psoriasis (PsO) samples were processed into annotated spot maps that formed the basis of the analysis (Figure 1A,B). PsO lesional (PsO-L) skin contained a higher proportion of γδT- (Figure 1C) and MAIT-enriched (Figure 1D) spots than PsO non-lesional (PsO-NL) and AD lesional (AD-L) samples, indicating selective expansion of both niches in established plaques. Within PsO-L, γδT-enriched spots showed higher fibroblast associated signatures (Figure 1E) whereas MAIT-enriched spots showed higher keratinocyte associated signatures (Figure 1F), indicating that the two iLTC niches occupy transcriptionally distinct tissue contexts within psoriatic lesions. All spatial transcriptomic analyses reflect γδT- or MAIT-enriched niches at the spot level and capture local microenvironmental transcriptional context, without resolving definitive cell-intrinsic functional states. Marker genes and lineage signatures supporting iLTC niche assignment are summarised in Supplementary Table S1. Transcriptional profiling further separated these niches (Figure 1G). γδT-enriched regions expressed markers consistent with a dermal sentinel like programme [6], including TCRγδ lineage genes (*TRDC*, *TRGC1*, *TRGC2*), cytotoxic and antigen presentation components (*KLRD1*, *B2M*) and features linked to tissue interaction and metabolic readiness (*EXTL1*, *RPL10*, *RPL13*, *MT CO3*, *ZNF569*) [7]. In parallel, MAIT-enriched regions displayed a transcriptional programme characteristic of tissue-positioned MAIT cells, prominently expressing *KLRB1* and *DPP4*—consistently highlighted in MAIT signatures from liver scRNA-seq [8], COVID-19 MAIT activation studies [9], and anti-mycobacterial response profiling [10]—alongside *CXCR6* and *IL7R*. These niches further upregulated transcriptional and metabolic features that support activation compatibility (*RORA*, *PIM1*, *GGH*, *ATP2C2*, *CSRNP1*, RNF222) [11,12,13]. Pathway enrichment reinforced this divergence, with γδT-enriched niches enriched for innate immune signalling and mitochondrial activity (Figure 1H) and MAIT-enriched niches enriched for epidermal development, barrier formation and lipid biosynthetic programmes (Figure 1I). These results show that γδT and MAIT niches are both expanded in psoriatic lesions and follow transcriptionally divergent tissue linked programmes.

### 2.2. Compartmentalised Epidermal–Dermal Organisation Distinguishes γδT from MAIT Niches in Psoriatic Lesions

**Figure 2:**
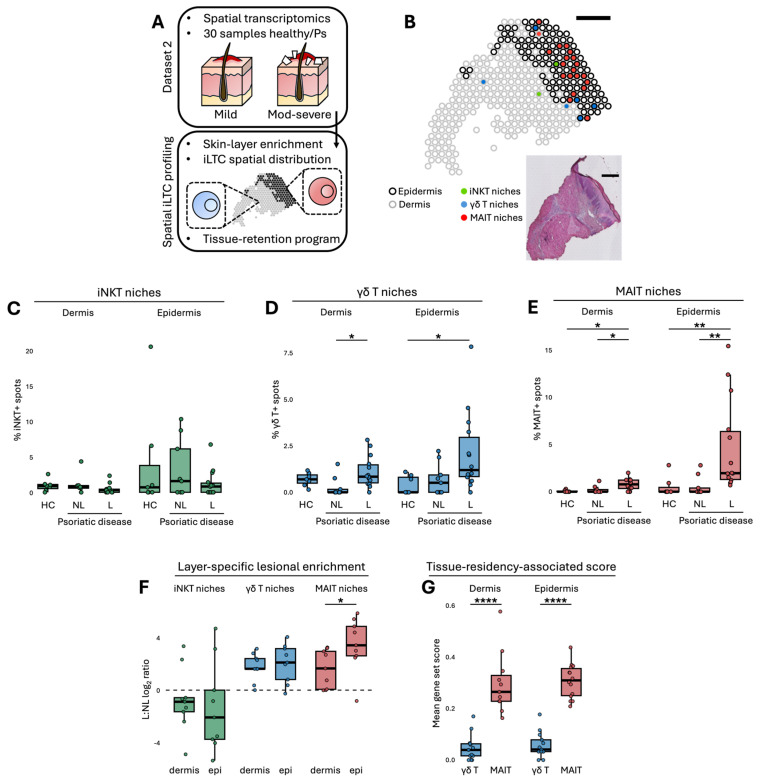
Spatial profiling reveals divergent layer-specific enrichment of innate-like T-cell niches in psoriatic skin. (**A**) Overview of the spatial transcriptomic analysis workflow. (**B**) Representative psoriatic sample showing epidermal and dermal segmentation with γδT, MAIT and iNKT signals. Scale bar = 500 µm. (**C**) Percentages of iNKT-, (**D**) γδT- and (**E**) MAIT-enriched spots in dermis and epidermis. (**F**) L:NL ratios of iLTC spot percentages. Comparisons between HC and Ps-NL epidermis did not reach statistical significance and are therefore not highlighted with significance symbols, consistent with the figure annotation throughout the manuscript. (**G**) Tissue-residency-associated scores. All analyses describe relative spatial enrichment across epidermal and dermal compartments. Kruskal–Wallis with Wilcoxon rank-sum tests were applied. Student’s *t*-test was used in panels (**F**,**G**). * *p* < 0.05, ** *p* < 0.01, **** *p* < 0.0001.

To validate the enrichment patterns observed previously, spatial transcriptomic sections from an independent cohort of healthy and psoriatic skin were analysed and used to resolve the layer specific organisation of iLTC niches through epidermal and dermal segmentation (Figure 2A,B). PsO and psoriatic arthritis (PsA) were jointly analysed as psoriatic diseases (Ps) to account for their shared cutaneous inflammatory features and to increase analytical power, following established integrative approaches in psoriatic research [14,15]. Quantification of iNKT-, γδT- and MAIT-enriched spots within each compartment showed that iNKT niche frequencies remained stable across healthy control (HC), Ps-NL skin and Ps-L skin in both layers (Figure 2C). Analysis of γδT-enriched niches revealed higher frequencies in Ps-L dermis relative to Ps-NL skin and were also increased in the epidermis compared with HC, with a substantial rise evident in both compartments (Figure 2D). Accordingly, subsequent analyses emphasise lesion-associated patterns, as comparisons with HC and Ps-NL skin did not reveal a consistent baseline epidermal enrichment of these niches. MAIT-enriched spots were similarly increased in Ps-L samples in both dermis and epidermis when compared with HC and Ps-NL, although the magnitude of increase was modest in the dermis and markedly higher in the epidermis (Figure 2E). Lesion to non-lesion ratios reflected these differences, with γδT showing comparable enrichment across layers whereas MAIT displayed a pronounced epidermal predominance (Figure 2F). Extending this divergence, MAIT-enriched niches displayed higher tissue-residency-associated scores than γδT-enriched niches in dermis and epidermis (Figure 2G). These analyses support the expansion of γδT and MAIT niches in psoriatic skin and show that their spatial organisation diverges across epidermal and dermal compartments.

### 2.3. Loss of Dermal γδT and Accumulation of Epidermal MAIT Niches Correlate with Psoriasis Severity

**Figure 3:**
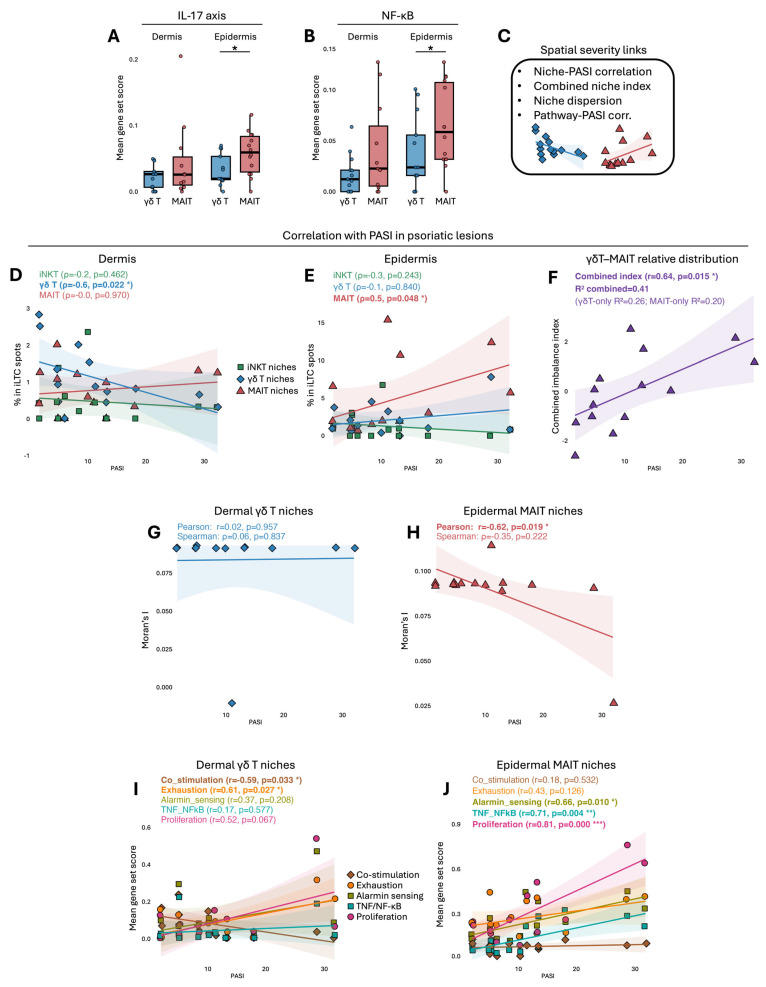
Psoriasis clinical scores align with opposing γδT and MAIT niche dynamics. (**A**) IL-17 response and (**B**) TNF/NFκB module scores in PsO-L niches. (**C**) Overview of the spatial transcriptomic analysis workflow. (**D**) Correlations between PASI and dermal γδT or (**E**) epidermal MAIT niche abundance. (**F**) Combined imbalance index (epidermal MAIT enrichment minus—γδT reduction) plotted against PASI. This index is included as a descriptive summary of opposing niche-level associations. (**G**) Moran’s I spatial autocorrelation of dermal γδT- and (**H**) epidermal MAIT-enriched niches relative to PASI. (**I**) PASI correlations with pathway module scores in dermal γδT- and (**J**) epidermal MAIT-enriched niches. Group differences in A-B were assessed by *t*-test when normally distributed or by Wilcoxon rank-sum test when not. * *p* < 0.05, ** *p* < 0.01, *** *p* < 0.001.

Given the distinct epidermal and dermal organisation of γδT and MAIT niches described above, inflammatory activity was first examined in Ps-L skin. *IL-17* and *NFκB* activity was increased in epidermal MAIT-enriched niches compared with epidermal γδT-enriched niches (Figure 3A,B), pathways that are central drivers of keratinocyte activation and chronic inflammation in PsO [1]. Subsequent analyses integrated iLTC niche features with cross-sectional clinical severity measures (Figure 3C). In lesional dermis, γδT-enriched niche abundance showed a negative correlation with PASI (Figure 3D), whereas epidermal MAIT niche abundance showed a positive correlation with PASI (Figure 3E). To summarise these opposing niche-level associations, a γδT–MAIT imbalance index was constructed as a descriptive summary metric to capture opposing associations between dermal γδT-enriched niches and epidermal MAIT-enriched niches (Figure 3F). This imbalance supports the interpretation that clinical trajectories across disease severity relate to tissue organisation of γδT- and MAIT-enriched niches along PASI-defined cross-sectional gradients rather than longitudinal disease progression. Spatial autocorrelation analysis showed that dermal γδT niches remained uniformly low and independent of PASI (Figure 3G), consistent with a scattered sentinel like distribution [6]. In contrast, epidermal MAIT niches showed progressively reduced clustering with increasing PASI, a pattern observed across lesions with heterogeneous epidermal architecture and inflammatory density indicating reduced spatial concentration across lesions with higher inflammatory burden (Figure 3H). These spatial metrics provide exploratory insight into how niche organisation varies across disease severity and should be interpreted as descriptive patterning within spot-level spatial data, given the resolution and sample size of the analysis. Pathway correlations showed that dermal γδT niches displayed a positive association between PASI and exhaustion-associated transcriptional modules, and a negative association between PASI and co-stimulation scores (Figure 3I). Epidermal MAIT niches instead showed positive associations between PASI and TNF and NFκB activity, proliferation related modules, and alarmin and IL-1 sensing signatures (Figure 3J). These modules reflect interlinked components of a coordinated inflammatory context, encompassing convergent cytokine signalling, transcriptional activation, and proliferative activity within a shared inflammatory environment. Together these findings indicate that clinical severity is accompanied by a shift from dermal γδT loss toward epidermal MAIT accumulation, with corresponding changes in spatial organisation and pathway activity.

### 2.4. Circulating γδT and MAIT Features Show Parallel Patterns Across the Psoriatic Spectrum

**Figure 4:**
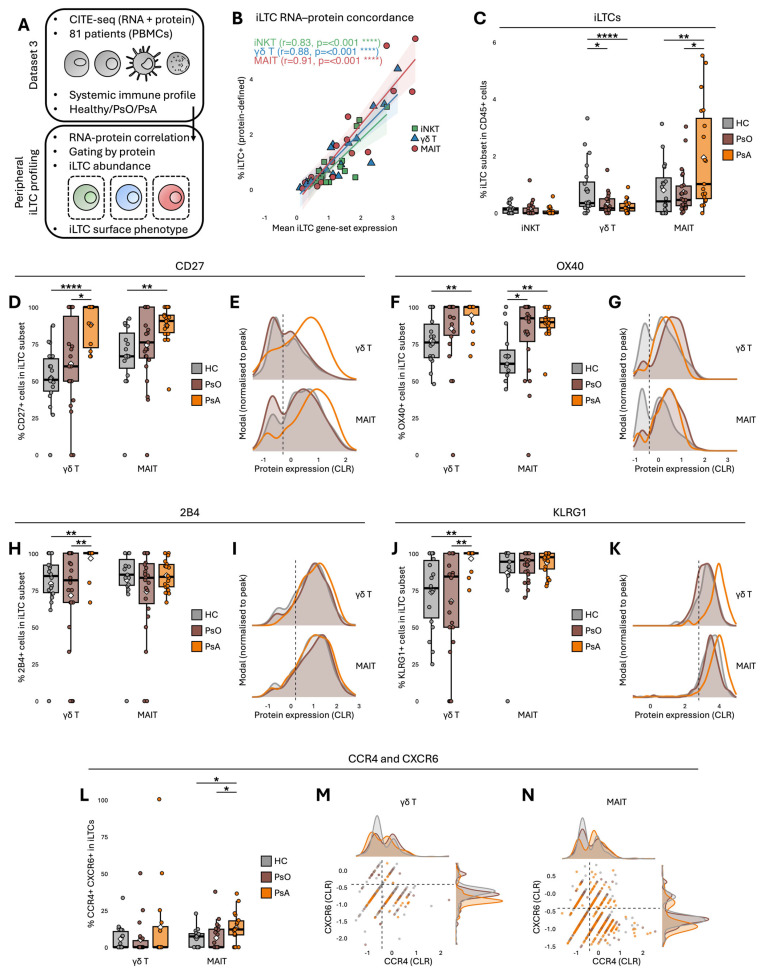
Systemic immune profiling reveals parallel γδT and MAIT alterations across the psoriatic spectrum. (**A**) Overview of the CITE-Seq analysis workflow. (**B**) iLTC RNA–protein correlations, with Spearman tests applied. (**C**) Percentages of iLTC+ cells within CD45+ events. (**D**,**E**) CD27+, (**F**,**G**) OX40+, (**H**,**I**) 2B4+, (**J**,**K**) KLRG1+, and (**L**–**N**) CCR4+ CXCR6+ cells within iLTC subsets. The dot colours in (**M**,**N**) correspond to the colour legend shown in the figure. Peripheral CITE-seq findings represent systemic immune alterations observed across the psoriatic spectrum, with prominent contributions from PsA, and are interpreted as parallel to tissue-associated patterns. Parametric (ANOVA with Tukey’s HSD) or non-parametric (Kruskal–Wallis with Wilcoxon rank-sum) tests were applied depending on data distribution. * *p* < 0.05, ** *p* < 0.01, **** *p* < 0.0001.

Building on the compartment specific divergences observed in skin, peripheral blood CITE seq data were examined to determine whether circulating iLTC subsets show corresponding patterns across the psoriatic disease spectrum (Figure 4A). Protein-based identification of iLTC subsets was performed using a CLR-normalised ADT gating strategy applied consistently across donors (Supplementary Figure S1). Surface defined frequencies derived from these gates showed strong concordance with iLTC gene set scores (Figure 4B). Circulating iNKT frequencies did not vary across disease groups (Figure 4C) and their protein based phenotypes were comparably stable across all markers examined (Supplementary Figure S2). Conversely, γδT proportions were reduced in PsO and PsA, whereas MAIT proportions were increased in PsA. These differences are consistent with the recognised immunological heterogeneity of PsA, which involves systemic immune activation and joint-associated inflammatory programmes that are not captured by cutaneous disease severity alone. Although derived from an independent cohort, these observations indicate that circulating γδT and MAIT subsets display disease-associated features that parallel selected aspects of their tissue-associated profiles. These parallels reflect shared inflammatory contexts across compartments rather than a direct skin-to-blood continuum driven solely by disease severity. These disease-associated alterations in circulating γδT and MAIT cell distributions were accompanied by increased CD27 and OX40 expression in both subsets in psoriatic disease compared with HC (Figure 4D–G), consistent with a shared antigen experienced and chronically engaged programme. Alongside this, 2B4 and KLRG1 increased specifically in γδT subsets in PsA (Figure 4H–K), indicating repeated stimulation and effector skewing rather than stable tissue residency. MAIT cells showed a complementary profile, with CCR4 and CXCR6 co expression increased in PsA (Figure 4L,M), matching their epidermal tropism and tissue retention profile observed in psoriatic plaques. Transcriptional features followed this trend, with RNA based pathway scores showing increased proliferation related activity in MAIT subsets in PsA, while other modules such as IL-17 axis, alarmin/IL-1 sensing and exhaustion displayed no major differences (Supplementary Figure S3). Together these findings indicate that γδT and MAIT subsets show distinct systemic patterns across the psoriatic disease spectrum, with the most pronounced differences observed in PsA, consistent with its higher inflammatory burden [16,17,18,19].

## 3. Discussion

Innate-like T cells occupy strategic tissue interfaces where rapid sensing, barrier interaction and immunological activation converge. In psoriatic inflammation, their contribution has been recognised but their organisation within lesions has remained incompletely defined. The present analysis shows that γδT and MAIT populations are expanded within psoriatic plaques, adopt distinct skin-layer arrangements, and engage transcriptionally divergent tissue-linked programmes. These layer-specific features relate to clinical severity through a shift from dermal γδT loss to epidermal MAIT accumulation, accompanied by changes in pathway activity. Peripheral profiling further reveals differences in γδT and MAIT subsets across the psoriatic spectrum, with the most pronounced alterations observed in PsA, indicating that systemic immune engagement co-occurs with aspects of the tissue-associated divergence.

The compartmental arrangement of γδT niches across psoriatic skin aligns with evidence that γδT niches adopt distinct behaviours in epidermal and dermal environments. Dermal γδT cells have been described as a heterogeneous and partly resident population with slow recirculation dynamics, expression of retention markers and the capacity to produce IL-17 and IL-22 in response to local inflammatory cues [20], supporting a sentinel role within stromal interfaces. Epidermal γδT populations follow a more homogeneous programme and represent a functionally distinct compartment in mouse models [21], indicating that γδT subsets interact dynamically with their surrounding tissue environments, with positioning reflecting a balance between intrinsic programmes and external signals. The broad distribution and transcriptional features observed here are consistent with this layered organisation and create a coherent framework for interpreting how other iLTCs relate to their own compartmental patterns.

In contrast to the wider distribution of γδT signatures, MAIT niches showed a strong epidermal predominance. Human studies indicate that MAIT cells preferentially localise to epithelial barriers where keratinocyte-derived cytokines, IL-7 availability, microbial metabolites and epithelial-homing receptors promote retention and activation [22]. Dermal MAIT cells appear considerably less frequent and are often characterised as more circulation-linked, with reduced engagement of barrier-associated pathways [23]. The marked epidermal bias observed in psoriatic lesions fits this broader pattern and suggests that epithelial conditions preferentially support MAIT accumulation and persistence. Taken together, these contrasts show that epidermal and dermal environments impose distinct constraints on γδT and MAIT programmes, shaping their divergence within inflamed psoriatic tissue.

The contrasting epidermal and dermal positioning of iLTC niches offered a structural framework for evaluating how these niches vary across increasing levels of clinical severity. Dermal γδT niches declined while epidermal MAIT niches became more prominent with increasing severity, and a combined imbalance index served as a descriptive summary of these opposing niche-level associations in cross-sectional tissue analyses, reflecting cross-sectional associations with PASI. Although the mechanisms underlying these changes cannot be resolved from transcriptomic data alone, the pattern aligns with reports that progressive psoriatic skin inflammation imposes metabolic and cytokine-driven pressures on the dermal microenvironment. These conditions can constrain the persistence of IL-17-competent dermal γδT cells, which experimental models characterise as partially resident, slow to recirculate, and central to neutrophil-rich inflammatory responses [24,25]. In contrast, the psoriatic epidermis becomes increasingly dominated by IL-17, IL-1 and TNF associated signals that favour the activation and retention of MAIT cells, matching observations that MAIT cells accumulate in epithelial barriers and contribute IL-17A in human psoriatic lesions [26]. The diffuse spatial distribution of MAIT niches in more severe plaques may therefore be interpreted in the context of epidermal expansion and architectural remodelling described in severe PsO, echoing descriptions of epithelial hyperplasia and barrier disruption in advanced PsO, where expanded cytokine responsive zones support broader positioning of unconventional T-cell subsets [1]. Together these observations suggest that increasing severity is accompanied by shifts in epithelial and stromal conditions that differentially sustain γδT and MAIT niches, placing distinct layer specific pressures on their persistence and functional potential.

Pathway analyses linked these anatomical patterns to distinct inflammatory contexts within each compartment. Dermal γδT niches showed increasing exhaustion-associated transcriptional features with rising PASI scores together with reduced co stimulatory features. These patterns reflect module-level activity within mixed cellular niches and are compatible with chronic inflammatory exposure rather than definitive functional exhaustion of γδT cells. Consistent with this interpretation, similar transcriptional shifts have been described in IL-23 and TNF driven models in which dermal γδT cells become less responsive under chronic cytokine exposure [20,24]. In contrast, epidermal MAIT niches exhibited stronger associations between severity and TNF and NFκB related activity, proliferative programmes and alarmin and IL-1 sensing pathways. These pathways are tightly interconnected and collectively describe an inflammatory milieu shaped by cytokine amplification, epithelial stress signalling, and activation within the psoriatic epidermis. Although these features require experimental validation, they align with the cytokine architecture of the psoriatic epidermis, where IL-1 family signalling, TNF amplification loops and keratinocyte hyperactivation maintain a state of heightened epithelial responsiveness [1]. MAIT cells integrate these cues through IL-7R, TNFR and innate sensing pathways, consistent with human observations that they display increased IL-17A production and proliferative activity in psoriatic plaques and other inflamed epithelial tissues [26]. Together these observations indicate that the molecular context of γδT and MAIT niches diverges with clinical severity, with dermal γδT niches showing signs of functional limitation and epidermal MAIT niches engaging pathways that reinforce their activation compatible behaviour within inflamed epithelial environments.

The distinct environments shaping γδT and MAIT niches within psoriatic skin were complemented by differences observed in circulation, most evident in PsA, a condition characterised by a higher systemic inflammatory burden than psoriasis alone [16,17,18]. Accordingly, PsA is considered here as an immunologically distinct disease entity rather than a linear extension of skin disease severity. As the peripheral and tissue datasets were derived from independent cohorts, these findings are interpreted as associative, reflecting parallel immune features across compartments without implying direct coordination along a blood–skin axis. Within this context, circulating γδT and MAIT frequencies showed patterns that mirror key aspects of their tissue-associated distributions. These systemic features should be interpreted as reflecting broader disease-specific immune states, particularly in PsA, rather than as evidence of severity-driven dissemination from skin to blood. Reduced γδT frequencies and increased MAIT frequencies in blood align with reports that systemic inflammation alters unconventional T-cell homeostasis and favours the expansion or mobilisation of cytokine responsive populations in psoriatic disease [27]. The increase in CD27 and OX40 expression across both subsets indicates chronic antigen experience [28,29], while the selective rise in 2B4 and KLRG1 within γδT cells in psoriatic arthritis is compatible with repeated stimulation and effector skewing described for chronically engaged γδT populations [30]. MAIT cells instead displayed increased CCR4 and CXCR6 co-expression, reflecting enhanced epithelial tropism and retention cues that parallel their strong epidermal engagement in psoriatic skin and align with evidence that CXCR6 is upregulated in MAIT cells during inflammatory states [31]. These systemic findings are consistent with the view that PsA involves broader inflammatory activation and suggest that circulating γδT and MAIT phenotypes reflect influences that also shape their behaviour within tissue.

Together these observations describe anatomically and transcriptionally divergent γδT- and MAIT-enriched niches in psoriatic skin, whose organisation across epidermal and dermal compartments associates with cross-sectional disease severity and parallel systemic immune features. The integration of spatially resolved transcriptomic maps with peripheral immune profiling provides a coherent view of how iLTC programmes unfold across tissue and blood in psoriatic disease, revealing parallel subset-level patterns across compartments. This layered framework highlights that γδT and MAIT niches respond to epithelial and stromal cues in distinct ways and that these differences are more pronounced across higher levels of inflammatory burden, offering a foundation for understanding how unconventional T-cell networks relate to the clinical spectrum of psoriatic pathology.

Some considerations frame the interpretation of these findings. Spatial transcriptomics captures spot-level rather than single-cell resolution, resulting in niche-level inferences that depend on the dominant transcriptomic profile within each spot rather than specific cellular contributions. Accordingly, γδT- and MAIT-enriched niches are defined by signature-driven dominance within mixed Visium spots, and do not represent definitive identification of individual cells. Given the relative rarity of these populations and partial transcriptional overlap between iLTC subsets, some degree of compositional ambiguity is unavoidable. The datasets analysed here sample representative but limited portions of lesional and non-lesional skin, and broader anatomical coverage may reveal additional patterns of iLTC organisation. Peripheral profiling provides complementary insights into systemic immune engagement but does not establish direct migratory or developmental relationships between blood and tissue compartments. Functional states and imbalance-associated mechanisms are inferred from transcriptomic and surface-marker signatures, and functional validation will be required to determine how the identified tissue programmes influence effector activity. In addition, spatial autocorrelation analyses are included as exploratory descriptors of niche patterning within sections and are constrained by spot-level resolution and sample size, limiting inference on fine-scale clustering or dispersion. These limitations do not detract from the overall framework but indicate that additional work integrating higher resolution spatial methods [32], longitudinal sampling and functional interrogation will be needed to refine the mechanistic links between γδT and MAIT niches, their layer specific properties and their contributions to psoriatic disease. Advancing these efforts will contribute to a deeper understanding of the immune architecture that characterises psoriatic lesions.

## 4. Materials and Methods

### 4.1. Data Sources

Gene Expression Omnibus (GEO) public datasets were retrieved and processed in R (v4.5.1) using Seurat (v5.3.0) [33]. The first spatial transcriptomics dataset (Dataset 1; GSE206391) [34] was used for iLTC mapping in AD and PsO skin samples. A scRNA-Seq dataset of HC and PsO skin (GSE162183) [35] supported leukocyte annotation. An independent Visium dataset (Dataset 2; GSE202011) [14,15], containing pre-annotated clinical severity scores was used to assess layer-specific enrichment and the association of iLTC co-localisation with local immune responses and disease severity. In addition, a CITE-seq dataset of peripheral blood mononuclear cells (PBMCs) (Dataset 3; GSE194315) [16] was used to assess gene–protein concordance, quantify iLTCs, and evaluate surface marker and gene signatures. As all datasets were previously published and public, no new samples were acquired or ethics approval required.

### 4.2. Spatial Transcriptomics

#### 4.2.1. Data Curation

Spatial transcriptomic datasets underwent standard processing and quality control to ensure cross-sample consistency and compatibility. Metadata and annotations were cross-checked against h5ad/Seurat objects and verified using Supplementary Materials to confirm accurate tissue–section correspondence. Quality control was performed at the spot level using library size, detected gene counts and mitochondrial transcript proportions, with dataset-specific field names harmonised prior to filtering. Sections meeting quality and completeness criteria were retained for downstream analyses.

#### 4.2.2. iLTC Niche Classification

Innate-like T-cell niches were defined using a combination of deconvolution-based lineage estimates and curated gene signatures listed in Table S1. Lineage-enriched compartments were first estimated by non-negative least squares deconvolution using the scRNA-seq reference. Deconvolution was performed at the Visium spot level, and relative lineage contributions were evaluated within each sample to account for differences in immune cell density and sequencing depth. Within the broader T-cell compartment, γδT, MAIT and iNKT niches were assigned based on dominant expression of their respective signatures relative to other T-lineage programmes and using adaptive per-sample thresholds designed to minimise bias from variation in immune-cell density. Because the canonical iNKT-defining α-chain transcripts were not represented in the Visium reference transcriptome for Dataset 1, iNKT niches were not assessed in that dataset. For all datasets, niches were defined at the Visium spot level, and per-sample frequencies of γδT-, MAIT- and iNKT-enriched spots were used for downstream comparisons across diseases, tissue layers and clinical phenotypes.

#### 4.2.3. Layer Compartment Classification

The regional classification from the original dataset was applied (GSE202011), based on clustering-supported histological alignment and marker expression. For clarity, histologically annotated regions were consolidated into a two-compartment model: keratinocyte-dominant strata (epidermis) and stromal-enriched regions (dermis).

#### 4.2.4. Differential Expression and Pathway Scoring

Differential expression analyses were performed using a two-sided Wilcoxon rank-sum test with Benjamini–Hochberg adjustment for multiple comparisons. Genes were considered significantly different when they showed consistent expression differences with a log-transformed fold change of at least 0.25 and an adjusted *p* value below 0.05, and were summarised in volcano plots highlighting the most strongly γδT-upregulated and MAIT-upregulated transcripts. Functional enrichment analysis was carried out using Gene Ontology Biological Process (GO:BP) categories, with enrichment significance assessed by a hypergeometric test with multiple-testing correction and pathways ranked by GeneRatio and adjusted *p* values. Pathway and module scores were calculated from curated gene sets compiled for this project and listed in Table S2. For each module, spot-level scores were defined from log-normalised expression values and aggregated to per-sample means within γδT and MAIT niches, stratified by dermis and epidermis where indicated. All downstream analyses were restricted to QC-passed spots and lineage-enriched niches as defined above.

#### 4.2.5. Severity Associations, Imbalance Index and Spatial Autocorrelation

Associations between iLTC niches and clinical severity were examined using the dataset annotated with PASI, the Psoriasis Area and Severity Index that quantifies lesion extent and inflammatory activity. For each psoriatic lesional sample, dermal and epidermal γδT, MAIT and iNKT niche proportions were correlated with PASI. To capture the opposing trends observed in psoriatic skin, a combined imbalance index was derived by subtracting the z-scored dermal γδT proportion from the z-scored epidermal MAIT proportion. This measure was compared with PASI alongside γδT-only and MAIT-only values. Spatial organisation of γδT and MAIT niches was assessed using Moran’s I computed on Visium spot coordinates within each layer. Resulting autocorrelation values were correlated with PASI to determine whether more clustered or dispersed iLTC architectures aligned with clinical severity. Linear fits shown on scatter plots were used as visual guides, with statistical associations based on rank correlations.

### 4.3. CITE-Seq

Raw RNA counts were log normalised and antibody-derived tag (ADT) values were normalised using centred log ratio transformation. Protein-level signals were further analysed in R using BCyto algorithms [36] adapted for multimodal protein measurements alongside Seurat-based single-cell processing.

### 4.4. Statistics

For each comparison, data distributions were first evaluated for normality using the Shapiro–Wilk test, and statistical tests were selected accordingly. When comparisons involved two groups, parametric (Student’s *t*-test) or non-parametric (Wilcoxon rank-sum) tests were applied depending on data distribution. For comparisons involving more than two groups, parametric (one-way ANOVA with Tukey’s post hoc test) or non-parametric (Kruskal–Wallis with Wilcoxon rank-sum post hoc tests) approaches were used, based on distributional properties. The specific tests applied in each analysis are indicated in the corresponding figure legends.

#### Declaration of AI Use

GPT-5 (OpenAI), a probabilistic language model based on statistical pattern recognition, was used to support manuscript preparation. All outputs were critically reviewed by the author to ensure factual accuracy and eliminate potential hallucinations.

## Data Availability

The data presented in this study are available in the Gene Expression Omnibus (GEO), accessible at https://www.ncbi.nlm.nih.gov/geo/ (accessed on 25 August 2025), at the following accession numbers: GSE206391, GSE162183, GSE202011, and GSE194315. These datasets were derived entirely from publicly accessible resources as detailed in Section 4.1.

## Supplementary Materials

The following supporting information can be downloaded at: https://www.mdpi.com/article/10.3390/ijms27020715/s1.

## Abbreviations

The following abbreviations are used in this manuscript:

AD	atopic dermatitis
ADT	antibody-derived tag
B2M	β2-microglobulin
CCR4	C-C motif chemokine receptor 4
CITE-seq	cellular indexing of transcriptomes and epitopes by sequencing
CXCR6	C-X-C motif chemokine receptor 6
GEO	Gene Expression Omnibus
GO:BP	Gene Ontology Biological Process
HC	healthy control
iLTC	innate-like T cell
iNKT	invariant natural killer T cell
IL	interleukin
KLRB1	killer cell lectin-like receptor subfamily B member 1
KLRD1	killer cell lectin-like receptor subfamily D member 1
KLRG1	killer cell lectin-like receptor subfamily G member 1
L	lesional
MAIT	mucosal-associated invariant T cell
NL	non-lesional
OX40	tumour necrosis factor receptor superfamily member 4
PASI	Psoriasis Area and Severity Index
PBMC	peripheral blood mononuclear cell
PIM1	proviral integration site for Moloney murine leukaemia virus 1
Ps	psoriatic disease
PsA	psoriatic arthritis
PsO	psoriasis
RORA	RAR-related orphan receptor alpha
RPL10	ribosomal protein L10
RPL13	ribosomal protein L13
RNF222	ring finger protein 222
scRNA-seq	single-cell RNA sequencing
TNF	tumour necrosis factor
TNFR	tumour necrosis factor receptor
TRDC	T-cell receptor delta constant
TRGC1	T-cell receptor gamma constant 1
TRGC2	T-cell receptor gamma constant 2
