# Correction: Bonilha, C.S. Spatial Imbalance of Innate-like T-Cell Niches Underlies Clinical Trajectories in Psoriasis. *Int. J. Mol. Sci.* 2026, *27*, 715

**DOI:** 10.3390/ijms27104401

**Published:** 2026-05-15

**Authors:** Caio Santos Bonilha

**Affiliations:** Institute of Infection, Immunity and Inflammation, University of Glasgow, Glasgow G12 8TA, UK; caio.bonilha@glasgow.ac.uk

## Remove Affiliations

In the published publication [1], there was an error regarding the affiliation for Caio Santos Bonilha. The affiliation “Institute of Developmental & Regenerative Medicine, University of Oxford, Oxford OX3 7TY, UK” was incorrectly included and should be removed. The correct affiliation is “Institute of Infection, Immunity and Inflammation, University of Glasgow, Glasgow G12 8TA, UK”. The author states that the scientific conclusions are unaffected. This correction was approved by the Academic Editor. The original publication has also been updated.
