# Supplementary material for: Spatial Imbalance of Innate-like T-Cell Niches Underlies Clinical Trajectories in Psoriasis
Source: Int J Mol Sci. 2026 Jan 10;27(2):715. doi: 10.3390/ijms27020715 (PMC12841213; doi:10.3390/ijms27020715)
Supplement: Supplementary file 1 [file ijms-27-00715-s001.zip › ijms-4030522-supplementary.pdf]

Table S1. Canonical marker genes used for compartment or cell classification.

Signature	Genes	Notes
**iNKT**	TRAV10, TRAJ18, ZBTB16	Canonical human iNKT TCR α-chain and transcription factor used to identify iNKT-enriched populations.
**γδT**	TRDC, TRGC1, TRGC2	Canonical γδ TCR chains used for identification of γδT-enriched spots.
**MAIT**	TRAV1-2, SLC4A10, CXCR6	Canonical MAIT markers used for MAIT spot identification.
**Fibroblast**	COL1A1, COL1A2, COL3A1, DCN, LUM, PDGFRA, FBLN1	Used to score stromal context in spot-level analyses.
**Keratinocyte**	KRT1, KRT5, KRT14, KRT6A, IVL, DSG1, FLG, LOR	Used to score epithelial context and epidermal positioning.

Table S2. Gene modules used for pathway analyses.

Module	Representative Genes	Functional Context
**Tissue-residency programme**	CD69, ITGAE, CXCR6, CCR6, CXCL16	Features associated with epithelial or stromal retention, reduced egress, and tissue positioning.
**IL-17 response**	IL17A, IL17F, CXCL1, CXCL2, CXCL8, DEFB4A, S100A8, S100A9	Keratinocyte-related inflammatory output and antimicrobial responses characteristic of psoriatic lesions.
**TNF/NFκB inflammatory**	TNFAIP3, NFKBIA, NFKBIZ, RELB, CXCL10, ICAM1, CCL20, IL1B	Core inflammatory pathways driving psoriatic cytokine signalling and stress responses.
**Co-stimulation**	CD80, CD86, ICOSLG, CD40, CD70	Co-signalling cues associated with T-cell activation and immune communication.
**Proliferation**	MKI67, TOP2A, PCNA, MCM5, MCM6	Cell-cycle progression and proliferative activity.
**Alarmin/IL-1 sensing**	IL1R1, IL1R2, IL1A, IL1B, S100A8, S100A9	Detection of epithelial alarmins and IL-1 family signals linked to barrier distress.
**Exhaustion**	LAG3, TIGIT, PDCD1, HAVCR2, TOX, ENO1, GAPDH, LDHA	Metabolic and inhibitory features associated with chronic stimulation or reduced effector capacity.

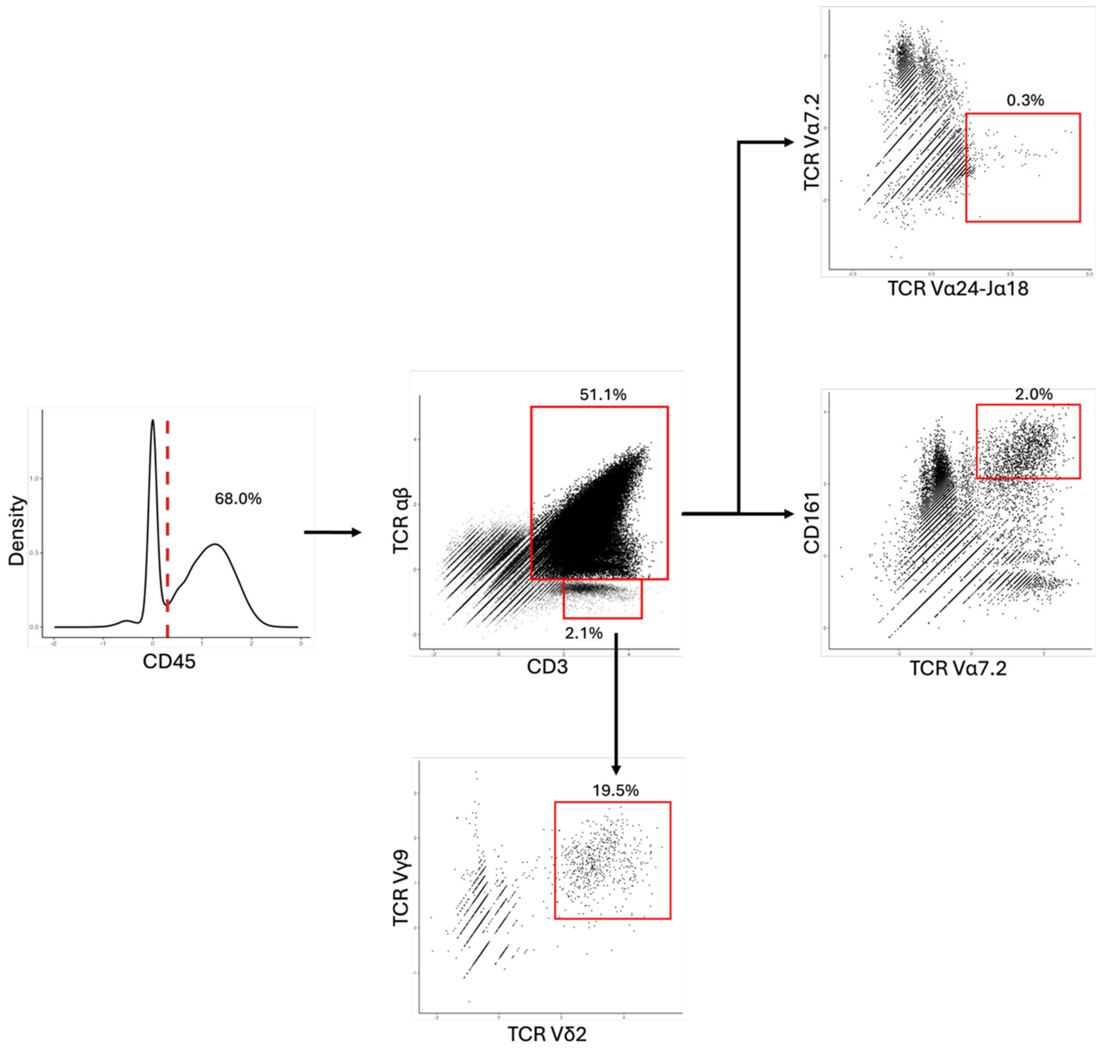

Supplementary Figure S1. Protein-based ILTC gating strategy for the CITE-seq analyses. Gating strategy applied to CLR-normalised antibody-derived tags (ADT) to identify innate-like T-cell subsets in PBMCs. CD45+ leukocytes were first selected. Within TCRαβ+ CD3+ cells, iNKT cells were identified as Vα24–Jα18+ Vα7.2- and MAIT cells as Vα7.2+ CD161+. γδT cells were defined as $CD3^{+}$ $TCR\alpha\beta^{-}$ $V\delta2^{+}$ $V\gamma9^{+}$. These gates were applied consistently across donors and used for all downstream abundance and phenotype analyses.

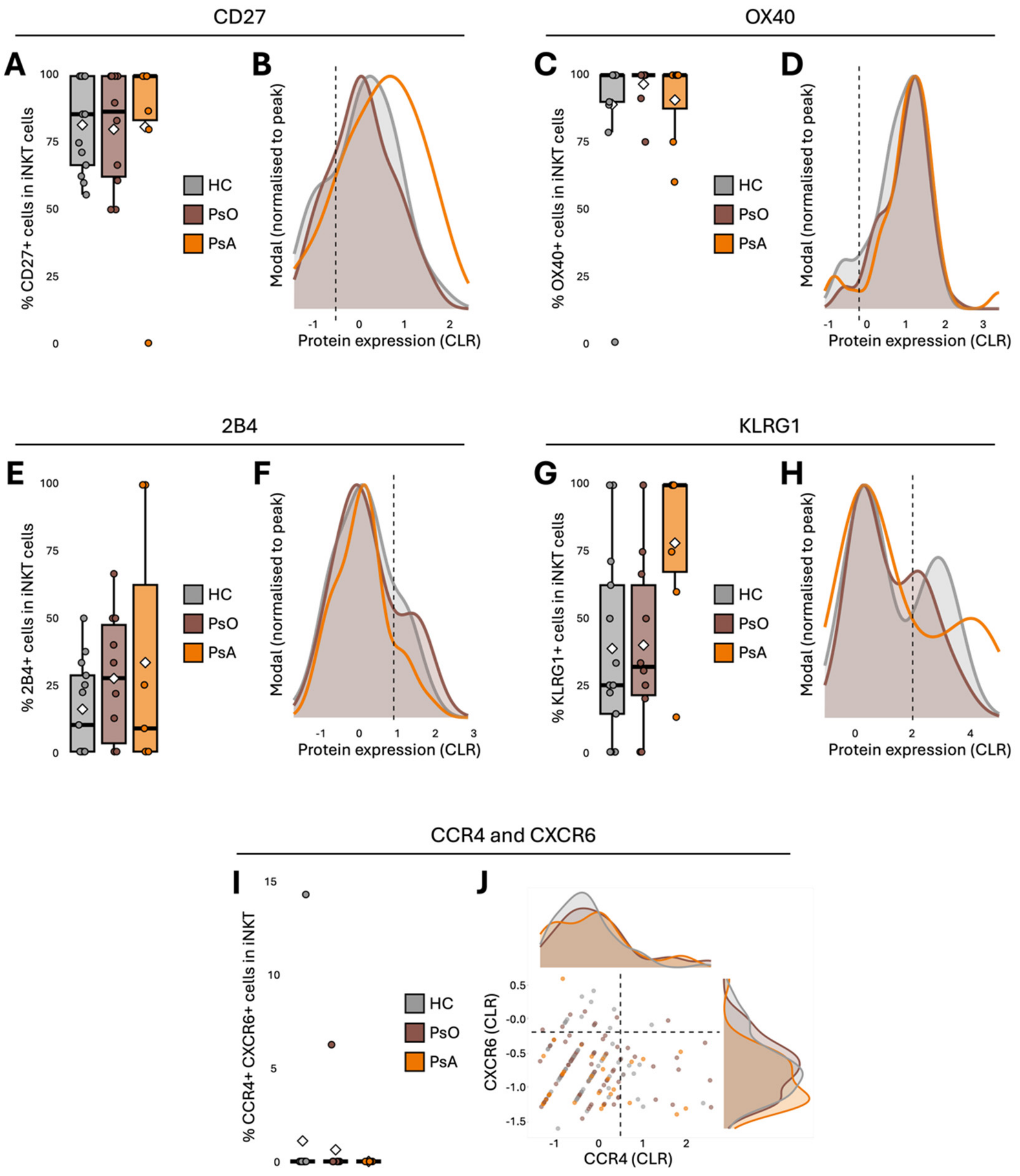

Supplementary Figure S2. Surface marker profiles in iNKT cells. (A-B) CD27+, **(C-D)** OX40+, **(E-F)** 2B4+, **(G-H)** KLRG1+, and **(I-J)** CCR4+ CXCR6+ cells within iLTC subsets. Parametric (ANOVA with Tukey's HSD) or non-parametric (Kruskal–Wallis with Wilcoxon rank-sum) tests were applied depending on data distribution.

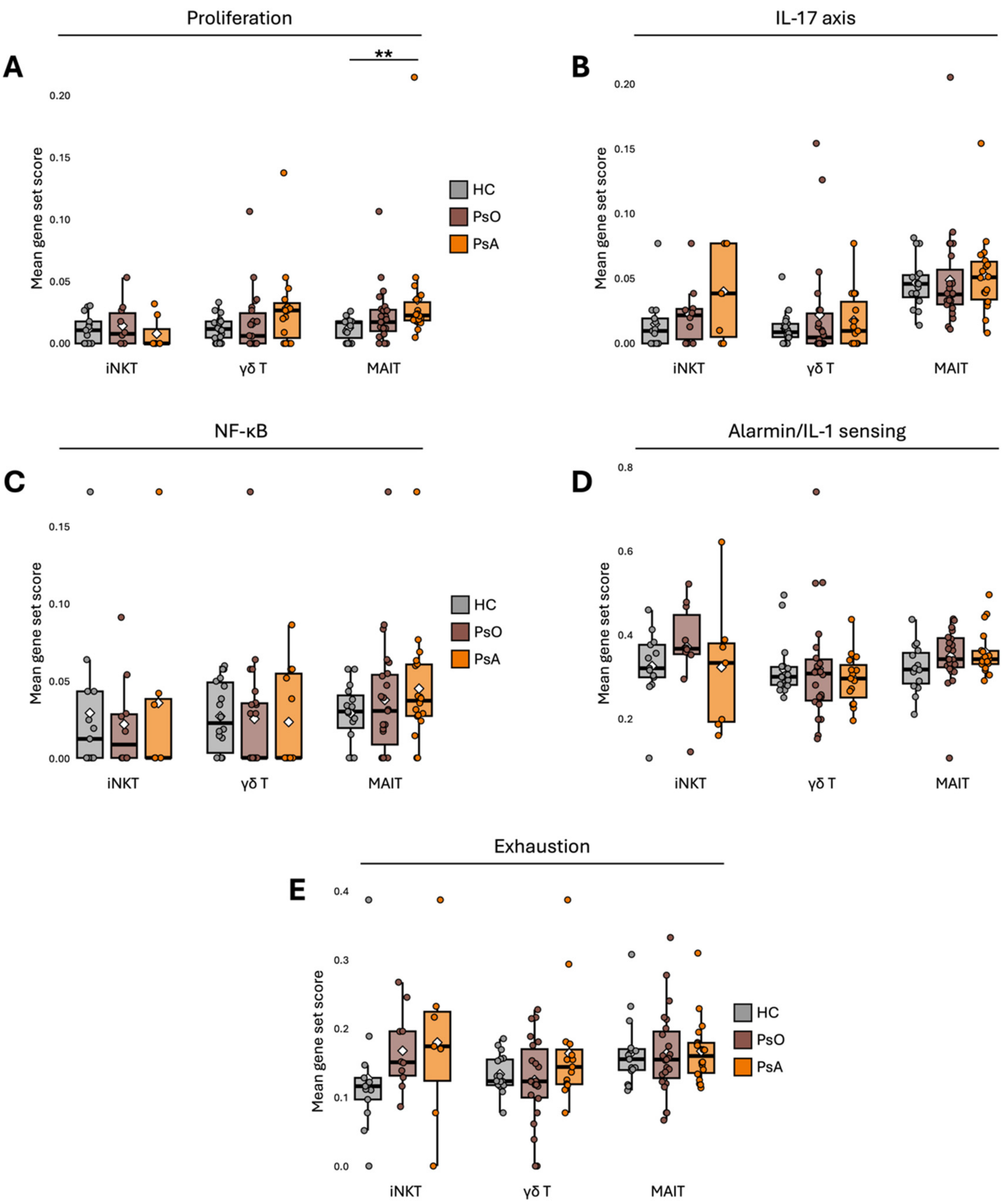

Supplementary Figure S3. RNA pathway scores in protein-gated iLTC subsets. (A) Proliferation, **(B)** IL-17 axis, **(C)** NFκB, **(D)** alarmin/IL-1 sensing, and **(E)** exhaustion module scores in iLTC subsets. Parametric (ANOVA with Tukey's HSD) or non-parametric (Kruskal–Wallis with Wilcoxon rank-sum) tests were applied depending on data distribution. **p<0.01.
